# Onchocerciasis-associated epilepsy and biomarkers

**DOI:** 10.1371/journal.pntd.0011808

**Published:** 2024-05-09

**Authors:** Robert Colebunders, Amber Hadermann, Alfred K. Njamnshi, Bruno P. Mmbando, Olivia Kamoen, Joseph Nelson Siewe Fodjo

**Affiliations:** 1 Global Health Institute, University of Antwerp, Antwerp, Belgium; 2 Brain Research Africa Initiative (BRAIN), Yaoundé, Cameroon; 3 Neuroscience Lab, Faculty of Medicine & Biomedical Sciences, The University of Yaoundé I, Yaoundé, Cameroon; 4 National Institute for medical Research, Tanga Research Centre, Tanga, Tanzania; 5 Department of Neurology, Heilig Hart Ziekenhuis, Lier, Belgium; Weill Cornell Medical College, UNITED STATES

There is increasing international recognition that onchocerciasis-associated epilepsy (OAE) is an important public health problem in onchocerciasis-endemic areas where onchocerciasis elimination programmes were either absent or functioning sub-optimally [1]. In several recent population-based studies, it was shown that OAE (which includes the nodding syndrome (NS)) is preventable by strengthening onchocerciasis elimination efforts [2–5]. However, the pathogenesis of OAE remains to be elucidated [6].

So far, little was known about the immunological processes associated with OAE. In a recent PNTD paper, Arndts et al. [7] describe the immune profile (cytokine, chemokine, and immunoglobulin levels) of persons with epilepsy including NS that were examined at the Mahenge epilepsy clinic in Tanzania between November 2014 and April 2015. Profiles of persons with epilepsy were compared with profiles of individuals without neurological disorders residing in the same endemic area. In all study participants, skin snips were collected and tested for *Onchocerca volvulus* presence using PCR. Study participants were classified into three groups: Persons with epilepsy (including NS)-*O*. *volvulus* PCR-negative, persons with epilepsy (including NS)-*O*. *volvulus* PCR-positive, and controls without neurological disorders-*O*. *volvulus* PCR-negative. Cytokines, most chemokines, and three neurodegeneration markers were comparable between *O*. *volvulus* infected and uninfected participants with epilepsy. However, elevated eosinophil percentages with increased eosinophilic cationic protein (ECP) and antigen-specific IgG levels were observed within the *O*. *volvulus*-positive epilepsy/NS group. In addition, the highest levels of the *O*. *volvulus*-related metabolic product (NATOG) were found in *O*. *volvulus*-positive NS persons.

We applaud the efforts of Arndts and collaborators to investigate an extensive panel of immunological markers in persons with epilepsy including NS. However, we recommend caution in drawing conclusions from their findings, since their study lacked a crucial group for comparison: onchocerciasis-infected individuals without any epileptic disorder. Moreover, they should have categorised the epilepsy/NS group into persons with OAE including NS and persons with other types of epilepsy to investigate whether they can be distinguished based on immune profile.

Mahenge area is an onchocerciasis-endemic area with a high prevalence of epilepsy, up to 3.5% in certain rural villages (8) with a high proportion of them (77.9%) meeting the criteria for OAE (9). In 2014–15, when the samples were collected at the Mahenge epilepsy clinic, most rural villages had no health facilities nor a constant supply of anti-seizure medication. Therefore, in order to obtain anti-seizure medication persons with epilepsy had to attend the Mahenge epilepsy clinic. In onchocerciasis-endemic areas with high *O*. *volvulus* transmission children first become O. *volvulus* infected and later develop epilepsy. This was shown in two cohort studies in Cameroon where a temporal and microfilaria dose-dependent association was observed between the level of *O*. *volvulus* infection in early childhood and the development of epilepsy later in life [8, 9]. The majority of participants in the *O*. *volvulus* PCR-negative epilepsy/NS group attending the Mahenge epilepsy clinic probably were persons with OAE who: (1) had been *O*. *volvulus* infected as young children; (2) developed epilepsy around the age of 8–12 years (the median age of the first seizures of persons with OAE) [10, 11] and (3) became *O*. *volvulus* PCR-negative due to prior ivermectin intake. In retrospect, indeed 37 (88%) of the 42 *O*. *volvulus* PCR-negative persons with epilepsy, for whom information was available, had taken ivermectin in the last five years.

The study findings of Arndts and collaborators suggest that most persons attending the Mahenge clinic presented a same type of epilepsy associated with onchocerciasis (OAE). Indeed, even persons with epilepsy who were PCR-negative presented with an *O*. *volvulus* immunological profile similar to PCR-positive persons with epilepsy. However, no OAE criteria were collected/presented, nor *O*. *volvulus* antibody testing was performed. Previous studies have shown that NATOG levels are increased in *O*. *volvulus* infected persons and that these levels correlate with the individual parasitic load [12]. The fact that the highest levels of NATOG were observed among *O*. *volvulus*-positive persons with NS was expected as NS is associated with a higher *O*. *volvulus* microfilarial load than other forms of OAE [11]. While determining NATOG levels may be useful in persons with epilepsy in high *O. volvulus* transmission zones, the NATOG test will have low discriminating power to differentiate between NS and other forms of epilepsy because most other forms of epilepsy in such areas will be OAE. In no or low *O*. *volvulus* transmission zones, there is no need for NATOG testing because NS is unlikely to occur. The elevated eosinophil percentages in the *O*. *volvulus* positive epilepsy/NS patients were also expected because increased eosinophil levels are associated with *O*. *volvulus* infection. There is currently overwhelming epidemiological evidence for the association between NS and *O*. *volvulus* infection [6]. In a recently conducted clinical trial in Uganda investigating the effect of doxycycline in persons with NS, 232 (96.7%) of the 240 persons with NS were found to present *O*. *volvulus* antibodies [13]. Considering the relatively low sensitivity of *O. volvulus* antibody testing, it is very likely that all persons with NS have previously been infected with *O*. *volvulus*.

The authors concluded that the biomarkers they identified might be useful for a differential diagnosis of epilepsy and nodding syndrome in *O*. *volvulus*-endemic areas. We disagree with this statement. While increased knowledge about neurodegenerative biomarkers and the patients’ immune profile may be of interest for the pathogenesis of OAE, these biological parameters do not seem to be useful to discriminate between OAE and other forms of epilepsy in onchocerciasis-endemic regions. Furthermore, the procedures to determine these biological parameters are complicated and difficult to implement as routine clinical or field research practice.

What is crucially important at the moment is a simple diagnostic approach or tool to differentiate OAE from other forms of epilepsy. Currently this is achieved by using the proposed OAE definition [14] which requires (1) good clinical history taking, (2) using epidemiological arguments, and (3) point-of-care onchocerciasis tests like the OV16 rapid diagnostic tests (or skin snip testing when possible). One major problem in overlapping *Taenia solium*- and onchocerciasis-endemic regions is to differentiate OAE from epilepsy caused by neurocysticercosis (NCC). The latter generally appears at an older age than OAE (beyond the age of 20 years) [15], though it would be useful to have a biomarker to precisely identify cases with epilepsy caused by NCC. Hereof, it has been suggested that individuals with a strong serologic response to *Taenia solium*, as evidenced by the appearance of four bands during the enzyme-linked immunoelectrotransfer blot (EITB) test, are likely to develop NCC-related epilepsy [16]. However, this approach is still unfeasible for resource-limited onchocerciasis-endemic settings.

On the other hand, more research needs to be conducted to identify the pathogenesis of OAE in order to enhance the specificity of potential diagnostic tools. To do so, we need to learn more about the biology of the *O*. *volvulus* worm and its vector, *Simulium*. A series of omics studies have recently been initiated including proteomics and viral metagenomics of the worm [6]. Such studies could potentially reveal specific biomarkers to differentiate OAE from other forms of epilepsy and from *O*. *volvulus* infection without neurological involvement.

During the sixth meeting of the WHO Onchocerciasis Technical Advisory Subgroup in December 2022, it was stated that there was convincing evidence for the association between onchocerciasis and epilepsy [1]. It was also proposed that WHO should organise a meeting involving neurologists, the WHO seizure and epilepsy group and onchocerciasis specialists to formalize recommendations for programmes regarding OAE [1]. In 2022, WHO also adopted the Intersectoral Global Action Plan (IGAP) for epilepsy and other neurological disorders [17]. In this plan the importance of infectious disease control for prevention of epilepsy is highlighted but onchocerciasis is not mentioned. Establishing an internationally accepted case definition of OAE would be useful for epidemiological studies, for programmatic purposes relating to brain health in highly endemic onchocerciasis foci, for monitoring/evaluating onchocerciasis national elimination programmes and for determining the true burden of disease caused by onchocerciasis.
